# Economic Evaluation of Fidaxomicin for the Treatment of Clostridium Difficile Infection (C. difficile-associated diarrhoea) in Ireland

**DOI:** 10.36469/9903

**Published:** 2015-05-06

**Authors:** Anke van Engen, Montserrat Casamayor, Fidelma Loftus, Martin Coen, Andy Garnham, Maureen Watt, Larry Lacey

**Affiliations:** 1 Quintiles, Hoofddorp, The Netherlands; 2 Quintiles, Barcelona, Spain; 3 Astellas Pharma Co. Ltd., Dublin, Ireland; 4 Astellas Pharma Europe Ltd, Chertsey, UK; 5 Lacey Solutions Ltd., Dublin, Ireland

**Keywords:** cost-utility analysis, macrocyclic antibiotic, recurrence, clostridium difficile infection, fidaxomicin

## Abstract

**Background:** Clostridium difficile is associated with 20–30% of cases of antibiotic-associated diarrhoea. The incidence of C. difficile infection (CDI) is higher in Ireland than in other countries in Europe, and it is associated with considerable morbidity. Previously recommended standard therapeutic options were vancomycin and metronidazole, but the macrocyclic antibiotic fidaxomicin has recently been recommended for use in adults with CDI in Ireland.

**Objectives:** To perform a cost-utility analysis of fidaxomicin compared to oral metronidazole (used to treat initial non-severe disease and first non-severe recurrence) and oral vancomycin (used to treat severe disease and any non-severe recurrence beyond the first) for the treatment of CDI.

**Methods:** A Markov model was used to determine the cost-utility of fidaxomicin in the treatment of all adult CDI patients (base case), patients with severe CDI and patients with initial CDI recurrences, respectively. Patients enter the model in the CDI health state and are treated either with fidaxomicin or current standard of care (oral metronidazole for non-severe CDI; vancomycin for severe CDI) for 10 days. The time horizon was 1 year. Deterministic and probabilistic sensitivity analyses were performed. Health state utilities were derived from the literature. The perspective was that of the Irish Health Service Executive (HSE).

**Results:** In the base case, fidaxomicin was dominant to current standard-of-care therapy, with cost savings of €2,904 and incremental quality-adjusted life year (QALY) gain of 0.031. The main drivers of costeffectiveness were recurrence rates and cost of hospitalization. Fidaxomicin was also dominant for all patient subgroups. The probability of fidaxomicin being cost-effective in all patients with CDI at a willingness to pay threshold of €45,000 per QALY gained was 82%.

**Conclusion:** Fidaxomicin was dominant to the current standard-of-care therapy for CDI. Based on this analysis, fidaxomicin has received reimbursement for CDI treatment under the High Tech Drug Scheme in Ireland.

## BACKGROUND

*Clostridium difficile* is associated with 20–30% of cases of antibiotic-associated diarrhoea, as well as the majority of cases of antibiotic-associated colitis and antibiotic-associated pseudomembranous colitis.[1] The most important risk factor for the development of *C. difficile* infection (CDI) is antibiotic use.[1-2] Advanced age and hospitalization are also recognised risk factors[1,3], with CDI affecting primarily the elderly and other frail subgroups such as immunocompromised, renal and cancer patients.

The incidence of CDI in Ireland is high[4-6], with a European hospital-based survey suggesting that the weighted mean incidence of CDI in Ireland, at 7.3 CDI cases per 10,000 patient days, was higher than that in Europe overall (4.1 CDI cases per 10,000 patient days).[7] A national overview of cases in Ireland in 2010 suggested that the incidence of CDI may be stabilizing or decreasing[8], but morbidity related to CDI remains considerable, with patients with CDI more likely than other patients to be discharged to a long-term care facility rather than to their home.[9] With increases in severity and frequency of occurrence in the community setting, CDI therefore remains a major public health concern.[4]

Before the introduction of fidaxomicin, the recommended standard therapeutic options for CDI were the glycopeptide antibiotic vancomycin and/or the nitroimidazole antibiotic metronidazole.[1,10] However, CDI recurs in approximately 20–25% of patients treated with vancomycin and metronidazole[8,11-14], and the probability of recurrence increases with each recurrence.[13,15] Therapies that reduce recurrence rates therefore have the potential to reduce the burden of CDI.

Fidaxomicin, the first in a new class of macrocyclic antibiotics[16], was developed with the aim of improving therapy by decreasing recurrence and complication rates, which contribute significantly to morbidity.[11,12,15,17] Two randomized double-blind phase III clinical trials demonstrated that fidaxomicin is non-inferior to vancomycin in terms of clinical cure and produces significant improvements in recurrence rates ($p<0.01$) and sustained cure rates ($p<0.01$).[18,19] Fidaxomicin is approved in the Republic of Ireland for the treatment of adults with CDI based on these trials[20], and the most recent CDI management guidelines in the Republic of Ireland recommend that fidaxomicin is used as an alternative to metronidazole or vancomycin for adult patients in various situations.[20]

We performed a cost-utility analysis of fidaxomicin treatment for adults with CDI compared to the standard of care: oral metronidazole for initial non-severe CDI and first non-severe recurrence; and oral vancomycin for severe CDI and any non-severe recurrence beyond the first, based on the European guidelines that were in use at the time the model was designed[10], although these have subsequently been updated.[21] This analysis was requested by the Health Service Executive (HSE) in Ireland during consideration of fidaxomicin for reimbursement under the High Tech Drugs (HTD) scheme.

## PATIENTS AND METHODS

### Analysis Populations

Fidaxomicin is indicated for all patients with CDI. The base case was a patient with CDI, irrespective of disease severity or whether the patient had a first CDI episode or a first CDI recurrence. The base case compared the use of fidaxomicin for all patients regardless of CDI severity to vancomycin for patients with severe CDI and metronidazole for non-severe CDI. The definitions of severe and non-severe CDI used in the model were those used to define mild and severe disease in the two phase III

trials comparing fidaxomicin and vancomycin.[18,19] However, based on discussion with the Irish National Centre for Pharmacoeconomics (NCPE), two separate scenario analyses were performed to assess the cost-effectiveness of fidaxomicin in patients with severe CDI and patients with first recurrence of CDI, because the reduction in the CDI recurrence rate may be of particular clinical relevance in these subgroups. For completeness, analysis of patients with non-severe CDI was also included.

**Pharmacoeconomic Model**

A Markov model was used to determine the cost-utility of fidaxomicin versus vancomycin and metronidazole in the treatment of all adult patients with CDI (base case) and the three additional patient subgroups (severe, non-severe, and recurrent). The basic model structure was based on the primary clinical endpoints of the clinical trials providing efficacy and safety data for the agents included in the model and current practice in Ireland (Figure 1).[18-20] The model included the health states shown in Table 1. In brief, patients enter the model in the CDI health state and are treated with fidaxomicin, oral metronidazole or vancomycin for 10 days. They can either live or die after having been infected with CDI; if patients survive, they can either be successfully treated (success) and go to the CDI cured health state, or fail to respond. Successfully treated patients remain in the CDI cured health state unless they die due to any cause (age-matched mortality) or experience a recurrence. In the case of a recurrence, they move back to the CDI health state and are retreated. Patients who do not respond to treatment may or may not have complications. To track the severity and number of recurrences, each health state (except the death health state) was split into five separate health states: index CDI episode; first non-severe recurrence; first severe recurrence; second or more non-severe recurrence; and second or more severe recurrence.

Cycle length was 10 days, corresponding to treatment course used in clinical trials and clinical practice.[10,20] The time horizon was 1 year because it was considered that this was sufficient to capture multiple recurrences; patients may have up to nine recurrences in 1 year but are unlikely to continue to recur beyond 1 year.[15] Therefore, discounting of healthcare costs and benefits was not required. The perspective was that of the Irish HSE; only direct healthcare costs were included.

**Model Inputs**

*Efficacy*

All efficacy data for fidaxomicin and vancomycin come from the protocol-specified modified intention-to-treat analysis set of two phase III trials, with similar study designs.[18,19] Both trials assessed clinical or symptomatic cure as the primary endpoint; recurrence and sustained cure rates were secondary[18] or exploratory[19] endpoints in these trials.

In the absence of a head-to-head clinical trial comparing fidaxomicin with metronidazole, an indirect treatment comparison[22] was undertaken based on a small single centre randomized, controlled trial comparing metronidazole (n=79) and vancomycin (n=71)[23] and the fidaxomicin studies.[18,19] This study[23] was identified by a systematic literature review to identify relevant studies in the treatment of CDI. All three studies included patients with severe and non-severe CDI, and proportions of these were similar in these three studies.[18,19,23] Differences between the trials included size (metronidazole study: n=150 versus fidaxomicin studies: n=1,101), where they were conducted (USA versus international), timing of the study (1994–2002 versus 2006–2009), and whether the *C. difficile* BI/NAP1/027 strain was a likely cause of CDI (0% versus 33.2/38.1%).

**Figure 1.** Model Structure (all CDI patients)

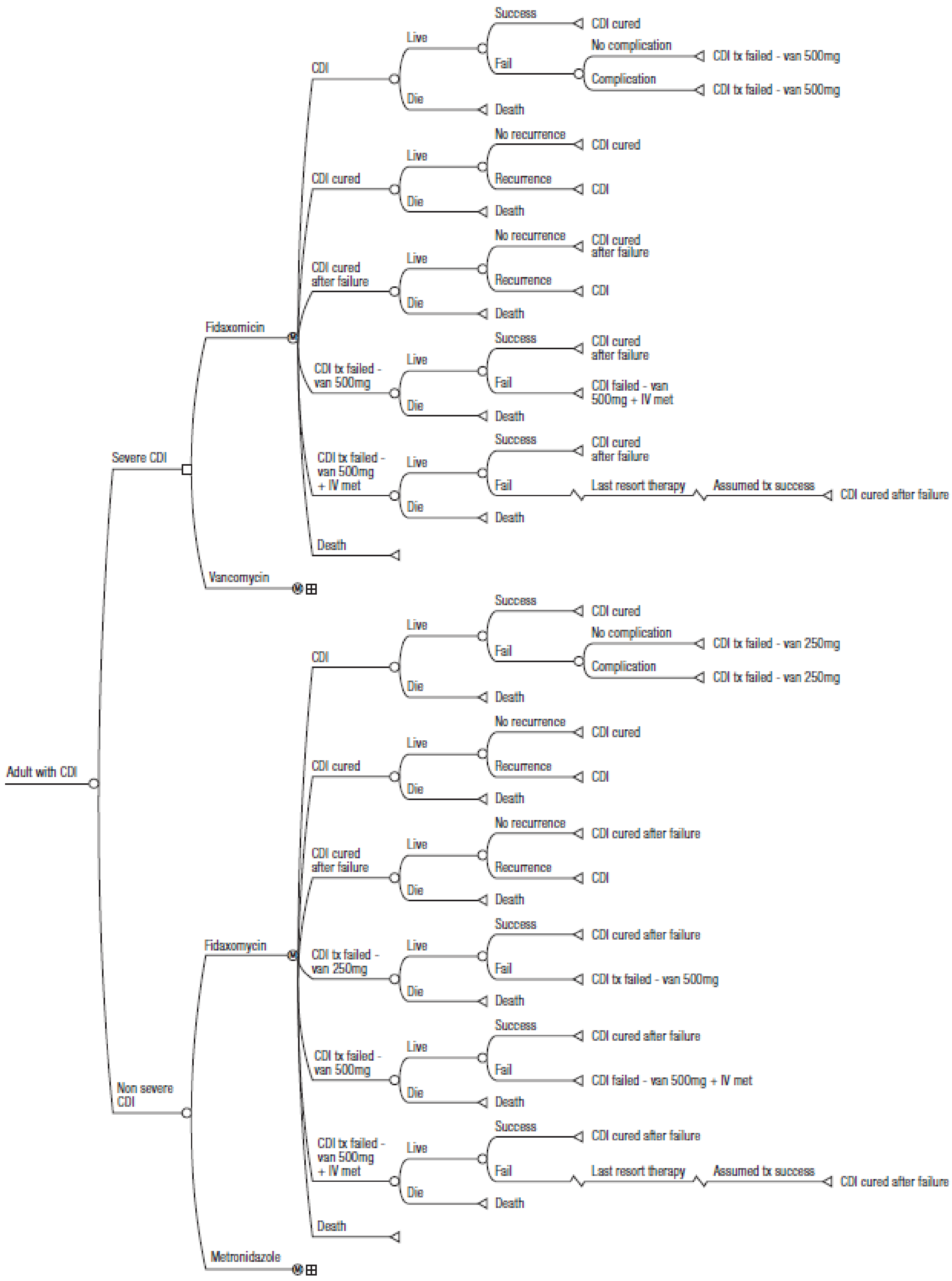

CDI: *Clostridium difficile* infection; IV: intravenous; met: metronidazole; tx: treatment; van: vancomycin

**Table 1.** Health States included in the Model

| Health State | Description |
|---|---|
| CDI[a] | The index CDI episode and any subsequent recurrent CDI episode; all patients enter the model in this health state |
| CDI cured | Patient clinically cured after initial treatment |
| CDI cured after failure | Patient clinically cured after initial treatment failure (in fidaxomicin arm only) |
| CDI treatment failed – vancomycin 250 mg | In case of non-severe CDI only: patient who failed to experience a clinical cure after 10 days of treatment with MTZ 400 mg TID and therefore receives treatment with vancomycin 250 mg QID for 10 days |
| CDI treatment failed – vancomycin 500 mg | Patient who failed to experience a clinical cure after 10 days of treatment with vancomycin 250 mg QID and therefore receives treatment with vancomycin 500 mg QID for 10 days |
| CDI failed – vancomycin 500 mg + IV MTZ | Patient who failed to experience a clinical cure after 10 days of treatment with vancomycin 500 mg QID and therefore receives treatment with vancomycin 500 mg QID + MTZ 400 mg TID (IV) for 10 days |
| Death | Patient who died due to either CDI or unrelated cause |

[a]Additionally, to keep track of the severity and number of recurrences, the CDI health state was split into seven separate health states: index CDI episode; first non-severe recurrence; first severe recurrence; second non-severe recurrence; second severe recurrence; third or more non-severe recurrence; and third or more severe recurrence.

CDI: *Clostridium difficile* infection, TID: three times per day; QID: Four times per day; IV: intravenous, MTZ: metronidazole

For the fidaxomicin versus vancomycin comparison, statistical heterogeneity was assessed by considering the chi-squared test for significance at $p<0.05$ or an I2 inconsistency statistic of ≥50% to indicate significant heterogeneity. For the clinical cure rate, marked heterogeneity was observed for severe (p=0.15, I2=51.91%) and non-severe CDI (p=0.09, I2=65.99%) based on the I2 inconsistency statistic; therefore, the odds ratio (OR) and its 95% confidence interval (CI) were calculated using the random effects model to provide a more conservative estimate of the effect. No heterogeneity was observed for recurrence rate or sustained clinical cure, and the OR and its 95% CI were calculated using the fixed effects model. ORs for all efficacy comparisons are shown in Table 2.

## Severity and Recurrence

A total of 12% of CDI cases were estimated to be severe, with 88% being non-severe [Astellas, data on file]. The CDI recurrence rate was 25%. The percentages of severe and non-severe CDI recurrence occurring within 30 days while in hospital were estimated to be 90% and 75%, respectively.

## Adverse Events

Drug-related adverse events for fidaxomicin or metronidazole or vancomycin were not included in the economic evaluation as they were generally mild in nature and were judged not to lead to additional treatment costs or, in general, to a switch in treatment.

**Table 2.** Efficacy Input Parameters

| Model Parameter | Base Case Input[a] | Range for Deterministic Sensitivity Analysis[a] |
|---|---|---|
| Odds ratio for clinical cure in non–severe CDI treated with fidaxomicin[18,19] | 1.450 | 0.630–3.360 |
| Odds ratio for clinical cure in severe CDI treated with fidaxomicin[18,19] | 0.858 | 0.502–1.465 |
| Odds ratio for clinical cure in CDI recurrence treated with fidaxomicin[18,19] | 1.078 | 0.415–2.808 |
| Odds ratio for clinical cure in non–severe CDI (first recurrence) treated with metronidazole[21] | 0.237 | 0.025–2.222 |
| Probability of clinical cure in non–severe CDI treated with vancomycin[18,19] | 0.878 | 0.802–0.897 |
| Probability of clinical cure in severe CDI treated with vancomycin[18,19] | 0.853 | 0.802–0.897 |
| Probability of clinical cure in CDI recurrence treated with vancomycin[18,19] | 0.889 | 0.817–0.945 |
| Odds ratio for recurrence in non–severe CDI treated with fidaxomicin[18,19] | 0.490 | 0.320–0.740 |
| Odds ratio for recurrence in severe CDI treated with fidaxomicin[18,19] | 0.456 | 0.264–0.788 |
| Odds ratio for recurrence in any CDI recurrence treated with fidaxomicin[18,19,b] | 0.528 | 0.256–1.086 |
| Odds ratio for recurrence in any CDI recurrence treated with metronidazole[23] | 1.639 | 0.257–10.369 |
| Odds ratio for recurrence with fidaxomicin compared to vancomycin for CDI patients with ≥2 recurrences[18,19] | 0.528 | 0.256–1.00 |
| Odds ratio for recurrence in patients with ≥2 previous recurrences[15] | 3.87 | 1.12–13.34 |
| Recurrence rate in non–severe CDI treated with vancomycin[18,19] | 0.231 (0.084) | 0.188–0.277 (0.067–0.102) |
| Recurrence rate in severe CDI treated with vancomycin[18,19] | 0.267 (0.098) | 0.205–0.333 (0.073–0.126) |
| Recurrence rate in patients with a recurrence treated with vancomycin | 0.325 (0.123) | 0.227–0.431 (0.082–0.171) |
| Recurrence rate after 30 days of end of treatment[18,19] | 0.084 (0.029) | 0.059–0.113 (0.000–0.392) |
| Probability of a complication with fidaxomicin or vancomycin (all CDI subgroups)[18,19,24] | 0.003 | 0.001–0.024 |
| CDI mortality (30–day)[25] | 0.060 (0.020) | 0.056–0.064[c] (0.002–0.002) |
| Annual all–cause mortality[26] | 0.013 (0.0004) | 0.000–0.090 (0.000–0.033) |

[a]Numbers in brackets refer to the calculated 10-day probability, where applicable.

[b]OR is the same as that for patients with their first recurrence at study entry.

[c]For the sensitivity analysis the maximum 30-day mortality rate for severe CDI was considered as 0.42 (0.105).

CDI: *Clostridium difficile* infection

## Hospital Length of Stay

The mean length of stay (LOS) attributable to CDI was based on data reported for St. James' Hospital, Dublin, Ireland, and was 22 days for severe CDI [NCPE, personal communication]. However, because attributable LOS depends on disease severity and whether patients experience a relapse, in the model LOS ranged from 10 days for non-severe CDI to 32.7 days for patients with severe CDI and a severe CDI relapse within 30 days of hospitalization. Attributable LOS of patients with severe CDI who were cured after 10 days and did not experience a recurrence was estimated to be 16.3 days, which compares with the 13 days estimated by Al-Eidan et al. in the only previous study in Ireland.[27]

## Costs

Drug costs included in the model are shown in Table 3. The base case assumes 25% community use, based on a survey of Irish microbiologists [Astellas, data on file].

Resource use was based on disease severity. Patients with severe CDI (index or recurrence) were assumed to be treated in hospital, whereas patients with non-severe recurrences were assumed to be treated in the community and to require two visits to a general practitioner. This is a conservative assumption based on a survey of Irish microbiologists [Astellas, data on file], which indicated that 5% of non-severe cases are treated in hospital. The base case analysis also took a conservative approach regarding the percentage of recurrences that are severe and set this at 12.2% based on data reported by Henrich et al.,[28] even though the incidence reported in the fidaxomicin studies was considerably higher.[18,19]

Costs of hospitalization (general ward: €871.74/day [range 697–1,046]; infectious disease ward: €1,026.00/day [range 820−1,231]), community treatment (GP visit: €28.00/visit [range 22.4–33.6]) and severe CDI complications (€19,670 [range 13,159–34,641]) were determined from a survey of Irish microbiologists [Astellas, data on file], the CASEMIX (2012 Ready Reckoner) and NCPE communications, respectively.

**Table 3.** Drug Costs per Course included in the Model

| Drug | Base Case Cost (€)[a] | Source |
|---|---|---|
| Fidaxomicin 200 mg BID for 10 days | 1,682.01 | Astellas |
| Metronidazole 400 mg TID for 10 days | 4.47 | MIMS Ireland (October 2011) |
| Vancomycin 250 mg QID for 10 days | 211.00 | NCPE |
| Vancomycin 500 mg QID for 10 days | 404.94 | NCPE |
| Vancomycin 500 mg QID + IV metronidazole 400 mg TID for 10 days | 409.41 | MIMS Ireland (October 2011) |
| Vancomycin taper regimen for 5 weeks | 218.00 | NCPE |

[a]All drug costs per course based on weighted average price of hospital and community use plus dispensing fee and assuming 10% wastage. Base case analysis assumes 25% community prescribing.

BID: Twice per day; TID: Three times per day; QID: Four times per day; IV: intravenous; MIMS Ireland: Monthly Index of Medical Specialties Ireland; NCPE: National Centre for Pharmacoeconomics Ireland

## Utilities

The health state utility estimate for CDI is based on estimates used in previous studies and was derived by Slobogean et al. (Table 4).[29] This is the only study that includes a derived health utility for CDI. The duration of impact of CDI on health-related quality of life (HRQoL) was assumed to be 10 days, i.e., the duration of therapy, but HRQoL was assumed to start to improve as soon as diarrhoea resolved. Complications were assumed to have a further disutility of –0.1.

**Table 4.** Health State Utility Values in Model

| Model Input Parameter | Index Episode and Severe Recurrence | Non-severe Recurrence |
|---|---|---|
| Utility for patients with CDI during first 10 days | 0.328 | 0.328 |
| Utility for patients for the first 10 days following cure | 0.560 | 0.780 |
| Utility for patients for the remaining period following cure | 0.780 | 0.780 |

CDI: *Clostridium difficile* infection

### Sensitivity Analyses

*Deterministic Sensitivity Analysis*

Extensive one-way analyses were performed to evaluate the parameters to which the model results were most sensitive and to test the robustness of the cost-effectiveness results. These were performed using realistic ranges for each parameter derived from the published sources of the base-case estimates (Table 2; online Appendix).

*Probabilistic Sensitivity Analysis*

Uncertainty surrounding the model input parameters was tested using probabilistic sensitivity analysis (PSA). The PSA was performed by recording the results of 10,000 Monte Carlo simulations, each of which simultaneously samples each of the model's input parameters (online Appendix). The results of the PSA are presented in the form of a graph displaying the results of the 10,000 simulations on the cost-effectiveness plane. A corresponding cost-effectiveness acceptability curve was calculated based on the results of the Monte Carlo simulations, with a focus on the willingness to pay (WTP) range of €20,000–45,000 per quality-adjusted life year (QALY) gained.

## RESULTS

### Cost-effectiveness

In the base case, fidaxomicin was dominant (more effective and less costly) compared to the current standard of care in the overall population of patients with CDI, resulting in cost savings of €2,904 and an incremental QALY gain of 0.031 (Table 5).

Similarly, treatment with fidaxomicin was associated with savings of €3,091 compared with metronidazole in non-severe CDI, of €1,588 compared to vancomycin in severe CDI, and of €4,650 compared to current treatment in patients with a first recurrence (Table 5). With incremental gains in QALYs, fidaxomicin was also dominant for each of these three patient subgroups (Table 5).

**Table 5.** Base Case Results – Mean ICER Over 1-year Time Horizon

| | Total Cost (€) | | | Total QALYs | | | |
|---|---|---|---|---|---|---|---|
| **Population** | **Current Care** | **FDX** | **Δ Cost (€)** | **Current Care** | **FDX** | **Δ QALYs** | **ICER (€)[a]** |
| All CDI | 23,591 | 20,687 | –2,904 | 0.695 | 0.726 | 0.031 | –94,128 |
| Non-severe CDI | 22,931 | 19,841 | –3,091 | 0.693 | 0.727 | 0.033 | –92,403 |
| Severe CDI | 28,337 | 26,779 | –1,588 | 0.707 | 0.719 | 0.012 | –128,335 |
| CDI Recurrence | 29,032 | 24,382 | –4,650 | 0.684 | 0.716 | 0.032 | –144,834 |

[a]Negative ICERs indicate that fidaxomicin is dominant, i.e., more effective and less costly than current care.

FDX: fidaxomicin; QALY: quality-adjusted life year; ICER: incremental cost-effectiveness ratio

## Sensitivity Analyses

### *Deterministic Sensitivity Analysis*

The results of the deterministic sensitivity analyses for all the assessed parameters which had an impact on the incremental cost-effectiveness ratio (ICER) are shown in Figure 2, which displays the impact of varying each individual parameter within its specified range on the ICER for all CDI patients. ICERs were highly sensitive to recurrence rates, and the model was most sensitive to the OR of experiencing a recurrence if patients had a history of ≥2 prior recurrences, for which the OR of experiencing a subsequent recurrence is 3.87 [1.12–13.34]; the OR of experiencing a recurrence with metronidazole; the OR of experiencing a recurrence with fidaxomicin in patients with a first recurrence; and the OR of a second or subsequent recurrence with fidaxomicin if patients had already had a first recurrence (Table 6). The other main driver of cost-effectiveness was reduced hospitalization costs with fidaxomicin (Figure 2).

**Table 6.** Threshold Analysis for Key Drivers of the Model

| | | All CDI Patients | |
|---|---|---|---|
| **Parameter** | **Base Case Value** | **Threshold Value (at ICER <€20,000)** | **Probability of Value below Threshold** |
| Odds ratio of experiencing a subsequent recurrence for patients with a history of ≥2 previous recurrences | 3.87 | 2.14 | 82.6% |
| Odds ratio of experiencing a recurrence with metronidazole | 1.00 | 0.74 | 95.4% |
| Odds ratio of experiencing a recurrence with fidaxomicin in patients with a first recurrence | 0.53 | 0.71 | 78.6% |
| Odds ratio of a second or subsequent recurrence with fidaxomicin in patients who have already had a first recurrence | 0.53 | 0.84 | 89.7% |

CDI: *Clostridium difficile* infection; ICER: incremental cost-effectiveness ratio

The results of a threshold analysis to identify the recurrence rate parameter values that would yield an ICER below a conservative threshold of €20,000 per QALY gained are shown in Table 6. The probabilities in favour of fidaxomicin were high in all cases. Taking the example of the risk of experiencing a recurrence in patients with a history of ≥2 prior recurrences, fidaxomicin remains cost-effective at a WTP threshold of €20,000 until the OR is <2.14, with the chance that the OR is >2.14 being 82.6%.

**Figure 2.** Tornado Diagram showing Results of the Deterministic Sensitivity Analyses

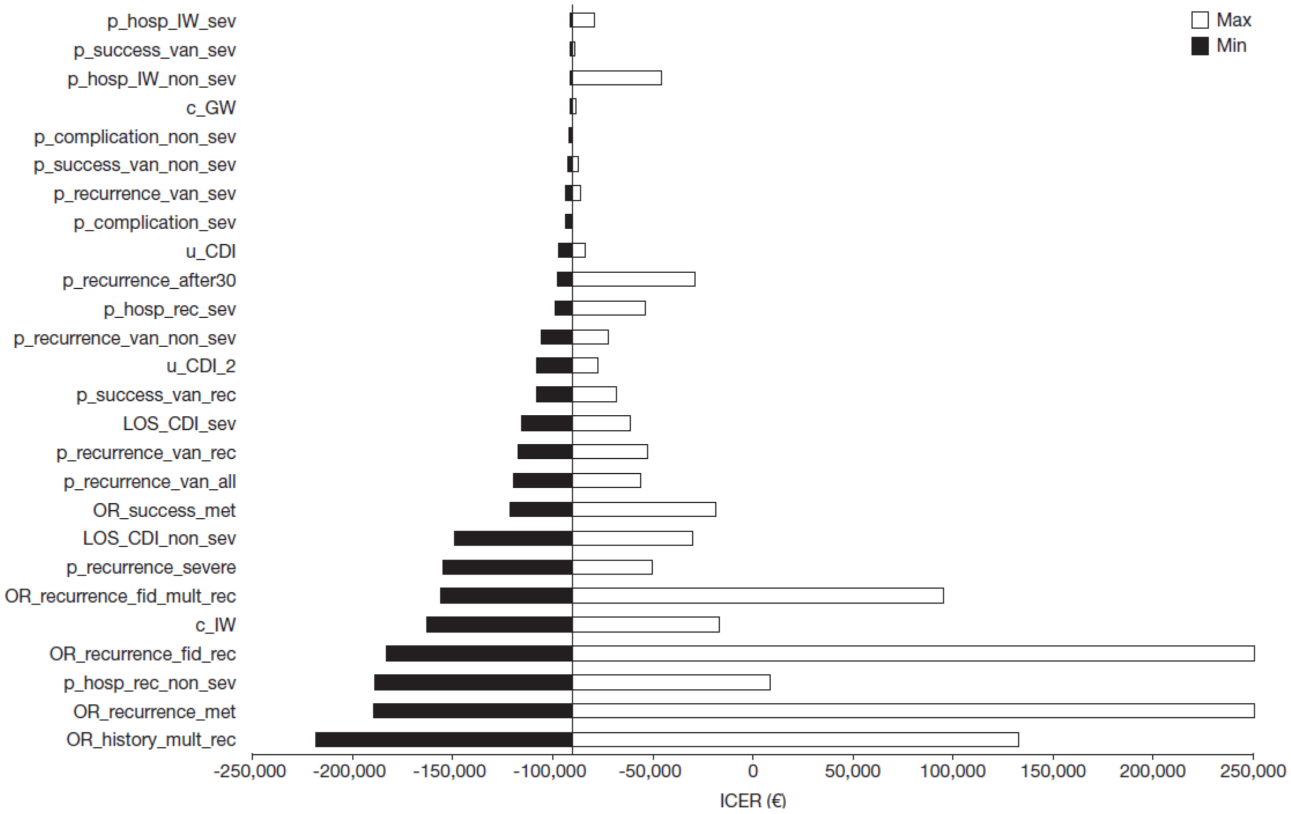

Note: The x-axis has been capped at €250,000 (where capped, maximum values were: OR_recurrence_fdx_rec, €809,801; OR_recurrence_mtz, €2,132,686).

ICER: incremental cost-effectiveness ratio; p_hosp_IW_sev: percentage of patients with severe CDI hospitalised on an infectious disease ward; p_success_van_sev: probability of clinical cure using VAN in severe CDI; p_hosp_IW_non_sev: percentage of patients with non-severe CDI hospitalized on an infectious disease ward; c_GW: cost per day in a general ward; p_complication_non_sev: probability of complications with non-severe CDI; p_success_van_non_sev: probability of clinical cure using VAN in non-severe CDI; p_recurrence_van_sev: probability of severe recurrence of CDI being treated with VAN; p_complication_sev: probability of complications with severe CDI; u_CDI: utility while having CDI; p_recurrence_after 30: probability of experiencing a recurrence after day 30; p_hosp_rec_sev: probability of a severe recurrence within 30 days of the previous episode being treated in hospital; p_recurrence_van_non_sev: probability of non-severe recurrence of CDI being treated with VAN; u_CDI_2: utility during the first 10 days of cure after severe CDI; p_success_van_rec: probability of clinical cure with VAN in recurrent CDI; LOS_CDI_sev: length of stay due to severe CDI (days); p_recurrence_van_sev: probability of recurrence with VAN in recurrent CDI; p_recurrence_van_all: probability of a recurrence with VAN in all patients; OR_success_met, odds ratio of clinical cure with MTZ compared to VAN; LOS_CDI_non_sev: length of stay due to non-severe CDI (days); p_recurrence_severe: probability of a severe recurrence of CDI; OR_recurrence_fid_mult_rec: odds ratio of a second or subsequent recurrence with FDX in patients who have had a first recurrence; c_IW: cost per day in an infectious disease ward; OR_recurrence_fid_rec: odds ratio of a recurrence with FDX in patients who have had a prior recurrence; p_hosp_rec_non_sev: probability of a non-severe recurrence being treated in hospital if occurring within 30 days of the previous episode; OR_recurrence_met: odds ratio of experiencing a recurrence with MTZ; OR_history_mult_rec: odds ratio of experiencing a further recurrence after ≥2 previous recurrences

*Probabilistic Sensitivity Analysis*

The PSA based on 10,000 simulations is provided in Figure 3. The mean incremental cost was –€4,171 (SD €4,799) and the incremental QALY was 0.023 (SD 0.014), demonstrating the dominance of fidaxomicin in all patients with CDI. The PSA demonstrated an 82% probability that fidaxomicin is cost-effective in all CDI patients at a WTP threshold of €45,000 per QALY gained (77% at a more conservative WTP threshold of €20,000 per QALY gained) (Figure 3).

**Figure 3A.** ICER Scatter Plot for all Patients with CDI

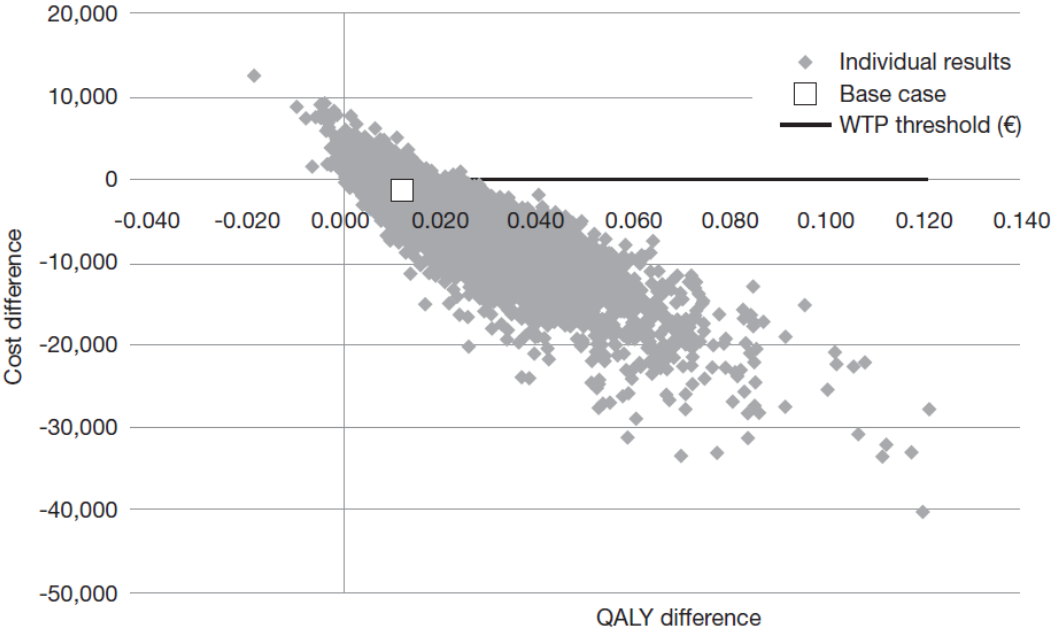

WTP: willingness to pay; QALY: quality-adjusted life year

**Figure 3B.** Cost-acceptability Curve for all Patients with CDI

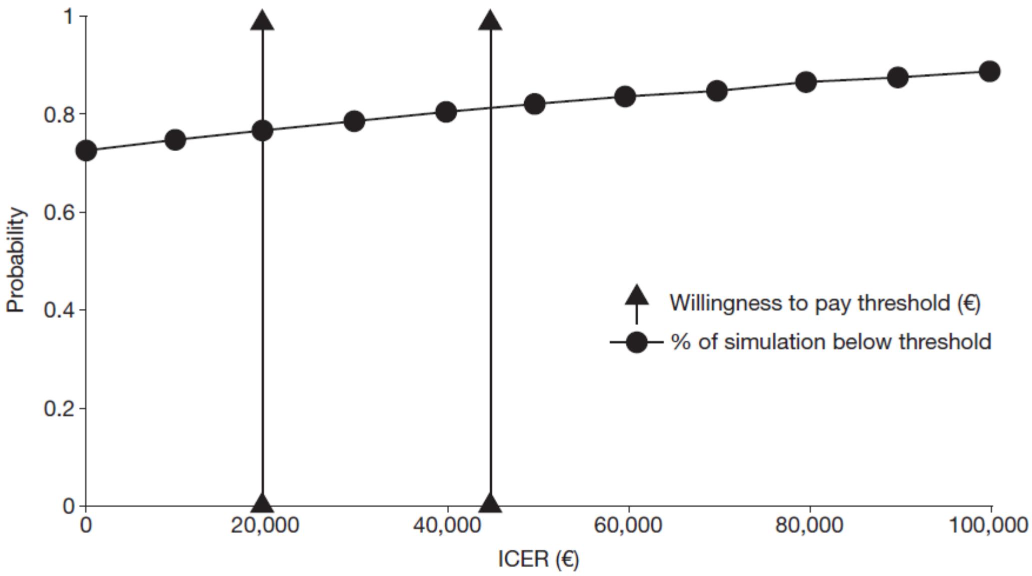

ICER: incremental cost-effectiveness ratio

## DISCUSSION

This analysis, which has been reviewed by the NCPE in the Republic of Ireland, examined the economic impact of fidaxomicin for the treatment of CDI from an Irish healthcare perspective. It demonstrates that fidaxomicin is dominant, i.e., less costly and more effective, compared to the standard of care for the treatment of CDI prior to fidaxomicin approval (vancomycin or metronidazole)[10], both in all patients with CDI and subgroups of patients with non-severe CDI, severe CDI and in patients with a first CDI recurrence. The cost-effectiveness of fidaxomicin therapy is driven mainly by reductions in the rate of recurrence of CDI. Deterministic and probabilistic sensitivity analyses were conducted to assess the robustness of the results. The deterministic sensitivity analyses showed that the probability of fidaxomicin being cost-effective was high across a range of variability in parameters, including recurrence rates. The PSA demonstrated an 82% probability that fidaxomicin was cost-effective in all patients with CDI at a WTP threshold of €45,000 per QALY gained.

Pharmacoeconomic analysis is an important element of drug assessment, particularly when new agents are more expensive than existing therapy and produce what may be perceived to be marginal gains in efficacy and/or safety. In the case of fidaxomicin, drug acquisition costs for a course of therapy are significantly greater than those of existing therapies (>€1,600 compared to approximately €200–400 for different doses of vancomycin and approximately €4.50 for metronidazole). However, drug acquisition costs form a relatively small proportion of the total costs of managing CDI, because hospitalization and complication costs are considerable (for a severe recurrence, €871.74/day on a general ward and €1,026 for a special infectious isolated room;[30] average weighted cost of a severe complication €19,180 based on CASEMIX 2012 Ready Reckoner). In the two clinical trials comparing fidaxomicin and vancomycin, fidaxomicin was non-inferior to vancomycin in terms of clinical cure, based on the lower boundary of the 97.5% CI being greater than –10% (87.7% versus 86.8% [lower boundary of 97.5% CI –4.9%] and 88.2% versus 85.8% [lower boundary of 97.5% CI –2.6%]); fidaxomicin has also been shown to produce significantly lower recurrence rates than vancomycin (12.7% versus 26.9% [p<0.001] and 15.4% versus 25.3% [p=0.005]) and significantly higher sustained response/global cure rates than vancomycin (76.6% versus 63.4% [p=0.001] and 74.6% versus 64.1% [p=0.006]) in these studies.[18,19] These results suggest that the efficacy of fidaxomicin is likely to have a significant bearing on whether it is a cost-effective option for the treatment of CDI. This was demonstrated by our analysis, which showed that the cost-effectiveness of fidaxomicin is due mainly to the reduction in the rate of recurrence.

Economic models generally have limitations related to the accuracy of the inputs. In our model, in addition to the trials comparing fidaxomicin to vancomycin[18,19], only one clinical study providing data on efficacy by disease severity was identified.[23] This randomised, double-blind, placebo-controlled study compared the efficacy and safety of metronidazole and vancomycin. No other study provided data by disease severity and a mixed treatment comparison was not feasible. The network meta-analysis conducted to compare fidaxomicin to metronidazole therefore used the methodology of Bucher et al.[31] It should be noted that differences in trial size, where and when they were conducted, and whether *C. difficile* BI/NAP1/027 was a likely cause of CDI may have led to a minor selection bias.

A related limitation of economic modelling is the need to make assumptions to address uncertainties, e.g., all patients are eligible for oral treatment or all vancomycin treatment is given as the oral solution formulation rather than as capsules. Assumptions may include estimates based on expert opinion, which can be influenced by bias. In our model, a number of assumptions were based on interviews with six microbiologists in the Republic of Ireland. The first related to the setting in which CDI of different severity is treated (hospital versus community); the second assumption was that complication rates in different treatment groups and different CDI populations were equal. The assumptions based on these interviews were generally conservative, e.g., approximately 5% of non-severe cases of CDI are treated in hospital, whereas we assumed that all non-severe cases are treated in the community. In addition, the six microbiologists interviewed represent 15−20% of the hospital-based clinical microbiology community in the Republic of Ireland, further improving the likely accuracy of the assumptions. Other assumptions related to the utilities for HRQoL in different disease states. While these utilities were based on published data,[29] assumptions were made about when patients would move between disease states.

Finally, estimates for model inputs are often uncertain. We addressed this by conducting two forms of sensitivity analysis. The deterministic sensitivity analysis varied input parameters to which the model was most sensitive across a realistic range defined using published sources. Fidaxomicin was generally cost-effective in these analyses. The PSA tested the uncertainty around parameter estimates and demonstrated that, even at a conservative WTP threshold of €20,000 per QALY gained, the probability of fidaxomicin being cost-effective was 77%; the probability was 82% at a threshold of €45,000 per QALY gained. Therefore, the sensitivity analyses demonstrate the robustness of the cost-effectiveness analysis performed. It should also be noted that the European guidelines used to determine the comparators for fidaxomicin in severe and non-severe disease in this analysis have subsequently been updated.[10,21] One of the key changes to the guidelines in terms of recommended therapy is the inclusion of fidaxomicin as an option for initial treatment of both non-severe and severe CDI and for the treatment of recurrent CDI.[21] This change is supported by our finding that fidaxomicin is more effective and less costly than the standards of care in the previous guidelines (metronidazole and vancomycin).[10]

## CONCLUSIONS

In conclusion, we have demonstrated that fidaxomicin is dominant to the previous standard of care for the treatment of CDI (vancomycin or metronidazole). The robustness of this pharmacoeconomic analysis of fidaxomicin is supported by the fact that it has been reviewed by the NCPE in the Republic of Ireland which led to fidaxomicin receiving reimbursement under the High Tech Drug Scheme. Furthermore, fidaxomicin has been included in the recently updated Irish CDI guidelines: as an alternative to metronidazole or vancomycin for adult patients with mild-to-moderate CDI; as an alternative to vancomycin for adult patients with severe CDI; in patients at high risk for recurrent CDI or with a first recurrence of CDI; and where concomitant antibiotics need to be used in patients with CDI.[20]

## ACKNOWLEDGEMENTS

We are grateful to the valuable input of Edward Crimin to the development of this analysis and earlier presentations of the data.

### Conflict of Interest Declaration

This work was supported by Astellas Pharma Co. Ltd., Dublin, Ireland. Medical writing support was funded by Astellas Pharma Europe Ltd. M.Coen is employed by Astellas Pharma Co. Ltd., Dublin, Ireland. F.L. was employed by Astellas Pharma Co. Ltd., Dublin, Ireland, when the analyses reported were undertaken. A.G. and M.W. are employed by Astellas Pharma Europe Ltd. L.L. is employed by Lacey Solutions Ltd., Dublin, Ireland, and A.E. and M.Casamayor are employed by Quintiles, both of which were contracted by Astellas to undertake the reported analyses. Medical writing support was provided by Andy Noble of Bioscript Medical.

## Figures and Tables

**Figure:** Supplementary Content

